# Cognitive Functions and Stereopsis in Patients with Parkinson’s Disease and Alzheimer’s Disease Using 3-Dimensional Television: A Case Controlled Trial

**DOI:** 10.1371/journal.pone.0123229

**Published:** 2015-03-30

**Authors:** Chan-Nyoung Lee, Deokwon Ko, Young-Woo Suh, Kun-Woo Park

**Affiliations:** 1 Department of Neurology, Korea University College of Medicine, Seoul, Korea; 2 Department of Ophthalmology, Korea University College of Medicine, Seoul, Korea; Sungkyunkwan University, KOREA, REPUBLIC OF

## Abstract

Stereopsis or depth perception is an awareness of the distances of objects from the observer, and binocular disparity is a necessary component of recognizing objects through stereopsis. In the past studies, patients with neurodegenerative disease (Alzheimer dementia, AD; Parkinson’s disease IPD) have problems of stereopsis but they did not have actual stimulation of stereopsis. Therefore in this study, we used a 3-dimensional (3D) movie on 3D television (TV) for actual stereopsis stimulation. We propose research through analyzing differences between the three groups (AD, IPD, and Controls), and identified relations between the results from the Titmus Stereo Fly Test, and the 3D TV test. The study also looked into factors that affect the 3D TV test. Before allowing the patients to watch TV, we examined Titmus stereo Fly Test and cognitive test. We used the 3D version of a movie, of 17 minutes 1 second duration, and carried out a questionnaire about stereopsis. The scores of the stereopsis questionnaire were decreased in AD patients, compared with in IPD and controls, although they did not have any difference of Titmus Stereo Fly Test scores. In IPD patients, cognitive function (Montreal cognitive assessment, MoCA) scores were correlated with the scores of the stereopsis questionnaire. We could conclude that Titmus fly test could not distinguish between the three groups and cognitive dysfunction contributes to actual stereopsis perception in IPD patients. Therefore the 3D TV test of AD and IPD patients was more effective than Titmus fly test.

## Introduction

Stereopsis, or depth perception, is one of the visual abilities to perceive the world in three dimensions, and enables humans to perceive close objects from distant objects. Stereopsis arises from a variety of depth cues, which are typically classified into monocular cues, and binocular cues. Monocular cues are represented in just two dimensions.[1] These cues include contrast,[2] relative size and height of the object,[3] texture gradients, motion parallax,[4] accommodation,[5] etc. Binocular cues include eye convergence, binocular disparity, etc.[6]

In order to perceive objects accurately, a sense of depth is needed that combines different scenes aroused by binocular disparity at the cerebral cortex. To perceive disparity, normal retina, visual acuity and proper alignment are basically needed.[6–8] A visual pathway that connects optic nerves to the lateral geniculate body, optic radiation, and visual striate cortex should also function normally. In addition to the visual striate cortex, the functions of other cerebral cortexes, especially the visual association cortex, are required to perceive objects. Tests on animals have shown that the visual association cortex, such as the second visual area (V2), systemizes binocular disparity.[9] Studies on fMRI (Functional Magnetic Resonance Imaging) also reported that visual association cortexes, such as V2, V3, V3A, V7, V4, V3A, and V7, are associated with stereopsis.[10,11] Motion perception is known to occur in V5 in the human visual cortex, suggesting that the visual association cortex is an important region in visual perception. Therefore, the perception of stereopsis requires integrated functions of eye and many parts of cerebral cortexes.

It is well known that patients with idiopathic Parkinson’s disease (IPD), or other neurodegenerative diseases, suffer from visual perception disorder, such as impaired visuospatial perceptions.[12–14] The IPD patient is particularly known to cause visual dysfunctions.^15, 16^ Studies that investigated binocular disparity in patients with IPD or other neurodegenerative diseases reported that their binocular disparity was weakened.[15–17] The causes of declined visual perception in these patients include: impaired nigro-striatal circuit; weakened light adaptation and decreased contrast sensitivity caused by declined dopamine cells in the retina; [18–22] and declined saccade and smooth pursuit eye movements.[23–25] In order to evaluate stereopsis, these studies analyzed parts of each monocular cue and binocular cue, respectively, and explained the role of the dopaminergic pathway—basal ganglion, corpus striatum, retina, etc.—relatively excluding the functions of the cerebral cortex. As explained above, since most of the monocular and binocular cues that enable humans to perceive stereopsis are controlled mainly by several parts of the cerebral cortex, we thought that a look into a cognitive function test that has close relations with the functions of the cerebral cortex may provide some insights.

Some studies that evaluated stereopsis in patients with IPD used the Titmus Stereo Fly Test and Random Dot Stereogram as evaluation tools.[26–28] Although these tests are common clinical tests for stereopsis, they measure stereopsis only by binocular disparity, without considering monocular cues, such as contrast, relative size and height of the object, texture gradients, motion parallax, accommodation, and binocular cues, like eye convergence. However, binocular disparity was not enough to measure actual stereopsis in the patients, because both monocular and binocular cues were needed to perceive stereopsis, giving rise to a need for new stereopsis stimuli, in order to measure actual stereopsis in the patients.

A recently commercialized 3-dimensional television (3D TV) displays offset images that are filtered separately to the left and right eye, to be combined by the brain, to produce 3D depth perception. Techniques employed for the television use both monocular cues and binocular cues, and produce 3D moving stimulations. Thus, using 3D television as an evaluation tool was thought proper to giving much actual and moving stimuli to IPD patients, in evaluating functions of their cerebral cortex. Since the mechanism of the 3D television test is distinct from those of the Titmus Stereo Fly Test and Random Dot Stereogram, it may provide a chance to analyze the differences of stimuli given by the 3D moving images, and the other two tests.

Alzheimer’s disease (AD) is one of the common neurodegenerative diseases that results in cognitive decline and weakened functions of several parts of the cerebral cortex, particularly the visuospatial function of the parietal lobe. Past studies have shown that declined perception of binocular disparity in AD patients resulted in weakened stereopsis, due to impaired functions of the cerebral cortex, such as the visuospatial function.[17,29] It may have been caused by a mechanism different from the mechanism that brings about declined functions of the substantia nigra and corticostriatal circuit, and weakened functions of dopaminergic neurons in the human retina, and eye movement disorder in IPD patients. Therefore, studying both AD patients and IPD patients in a stereopsis study that uses 3D TV may produce results that allow analysis on how cognitive function affects actual and moving stereopsis.

This study compared and analyzed the survey results from IPD, AD patients and normal people who went through experiments—the Titmus Stereo Fly Test, and a test that used 3D TV—that measured their stereopsis. The survey revealed differences between the three groups, and identified relations between the results from the Titmus Stereo Fly Test, and the 3D TV test. The study also looked into: factors that affect actual stereopsis; how cognitive function affects stereopsis; and what it means to use a 3D TV, in measuring stereopsis in patients with degenerative diseases.

## Methods

### Ethics Statement

Each participant was informed about risks and inconveniences associated with the experiment. All subjects gave written informed consent. This study was approved by the Institutional Review Board, at Korea University Anam Hospital and met the standards of the Declaration of Helsinki.

### Inclusion and exclusion criteria for the study population

In order to measure stereopsis in patients with degenerative diseases, we recruited an IPD patient group, an AD patient group, and a control group with no neurological deficits, who visited the department of neurology at Korea University Anam Hospital. 52 IPD patients and 39 AD patients were selected, based on the Queens Square Brain Bank criteria, and NINCDS-ADRDA criteria respectively.^34, 35^ 32 normal people were selected for the control group. Patients with strabismus, nystagmus, ocular motility disturbance, and poor visual acuity in either eye (< 20/40 Snellen fraction) were excluded from the study sample. IPD and AD patients who scored more than 800 arc seconds (arcsec) in the Titmus Stereo Fly Test were excluded, as they were deemed unable to watch 3D TV. Illiterate patients, or patients who got scores below 11 points in MMSE (Minimal Mental Status Examination), were also ruled out, as they were unable to read, or to perceive things properly. IPD and AD patients were told to continue taking their medications. We received informed consent from all participants. This study was approved by the Institutional Review Board, at Korea University Anam Hospital.

### Experiments

In addition to checking the gender and age of IPD and AD patients, MoCA (Montreal Cognitive Assessment) and MMSE that assess several cognitive domains and the Clinical Dementia Rating (CDR) were conducted on the patients, to evaluate their basic conditions.[30–32] But the normal control group underwent only MoCA. The Hoehn and Yahr (H&Y) stages of the IPD patients were revealed, to show the severity of their symptoms.[33] Those who did not meet the experimental criteria were excluded from the groups afterwards, through ophthalmic and neurological exams, and a binocular disparity exam (the Titmus Stereo Fly Test).

3-D images were shown through a 55-inch film patterned retarder 3D TV, with a 2.7 meter viewing distance, as recommended by the TV manufacturer. The same degree of brightness and sound were maintained in the room, with the same intensity of illumination, while MBC’s drama “Gaebak” was shown for 17 minutes and one second. Horizontal side-by-side type stereoscopic images were used for the test. Viewers who were advised to pay attention to the film wore eyeglasses, which contained a pair of polarized filters.

After watching the drama, an investigator explained the questions, and viewers answered the questions. The questions asked viewers how three-dimensional the images looked to them. They were told to score 5 if they felt all images looked like 3D images during the whole viewing time. If some backgrounds or objects were not stereoscopic, they were told to give 4. 3 points were given, if the large objects in the scene were not stereoscopic, 2 when viewers saw nothing but some moving objects that were drawing closer to them, and 1 when the scene itself was not stereoscopic at all. (1: the scenes were not stereoscopic at all ~ 5: no problem in seeing stereoscopic images). We used the stereopsis questionnaire which had the form of visual analog scale (VAS). Scores were evaluated, based on the above standard.

### Statistics

Answers to the surveys about demographic factors, cognition test, corrected eyesight, and stereopsis of the people in the three groups—IPD patient group, AD patient group and normal control group—were compared, based on the Mann-Whitney U test, when the two groups were compared; but ANOVA was used, when all three groups were compared together. In order to analyze the results from the Titmus Stereo Fly Test, we assumed that those who scored below 60 arcsec were normal, while those who scored more than 60 arcsec were abnormal in their binocular disparity, because 60 arcsec is determined as normal in the Titmus Stereo Fly Test. Then, the answers were analyzed by Pearson’s chi-square test. Pearson’s correlation was used to find correlation among ages, MoCA, scores of stereopsis questionnaire, and Titmus Stereo Fly Test figures. Spearman’s correlation (partial correlation) was used to analyze partial correlations among ages, and total scores of MoCA and stereopsis surveys. SPSS for Windows 15.0 (SPSS Inc., Chicago, IL, USA) was used to analyze the study results, and those with p-value<0.05 were classified as having statistical significance.

## Results

A total of 123 people (52 IPD patients, 39 AD patients, and 32 normal people) participated in the experiment. 3, 2, and 3 people were excluded from the IPD group, the AD group and the control group, respectively, as their Titmus Stereo Fly Test scores went over 800 arcsec. 3 people had strabismus, 2 had cataract, 2 had low visual acuity and 1 is unknown cause. Male patients accounted for 46%, 37% and 38% of each group, respectively, with no statistical significance. The ages of the AD group were statistically significant—they were about 5 years older than the other two groups. AD patients scored statistically significant lower points in MMSE, than did IPD patients. AD patients scored the least points in MoCA, followed by IPD patients, and the normal control group. Most of the AD patients were classified as those in an early stage of dementia, with CDR scores of 1.08±0.28. Most of the IPD patients were classified as patients in the early and middle stages of IPD, with H&Y stage scores of 2.35±0.86 (9 people were in stage 1, 1 person in stage 1.5, 12 people in stage 2, 9 people in stage 2.5, 14 people in stage 3, and 4 people in stage 4). No statistically significant difference was found in the eyesight of those in the three groups (Table 1).

**Table 1:** Demographics, clinical data of idiopathic Parkinson disease patients, Alzheimer’s disease patients, and Controls.

	IPD	AD	Controls	*P*-value
Sex (men: women)	18: 31	17: 20	11: 18	NS
Age*	67.27±9.63	73.73±6.79	68.14±7.47	*P* = 0.001^a^, NS^b^, *p* = 0.020^c^
K-MMSE score	25.41±3.27	21.03±3.51	-	<.001
MoCA score*	21.49±5.39	14.86±4.37	24.48±2.91	*P*<0.001^a^, *p* = 0.016^b^, *P*<0.001^c^
CDR score	0.57±0.26	1.08±0.28	-	<.001
H&Y stage	2.35±0.86	-	-	NA
Corrected Visual acuity (Left)*	0.75±0.25	0.57±0.27	0.73±0.32	NS
Corrected Visual acuity (Right)*	0.76±0.25	0.55±0.26	0.75±0.30	NS

NS: Not significant, NA: Not analyzed, AD: Alzheimer’s disease, IPD: Idiopathic Parkinson’s disease.

* Statistical tests: ANOVA

^a^: IPD vs AD

^b^: IPD vs Controls

^c^: AD vs Controls

### Results of the Titmus Stereo Fly Test

**Fig 1:**
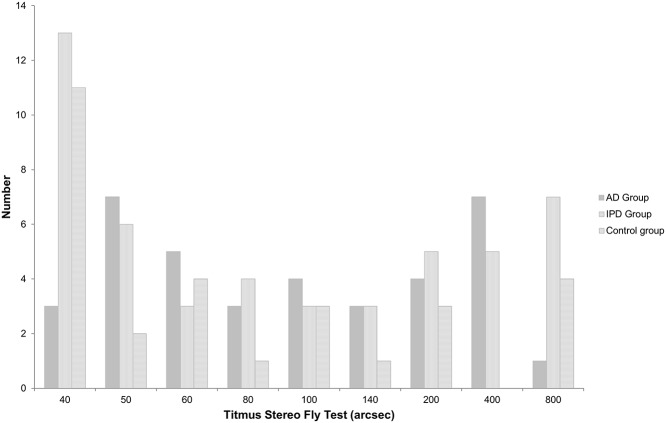
The number of patients, according to the Titmus stereo Fly Test, in three groups. IPD: Idiopathic Parkinson’s disease, AD: Alzheimer’s disease.

Patients in each group were classified, and are presented, by their arcsec scores in the Titmus Stereo Fly Test (Fig 1). The number and proportion of normal and abnormal people in the IPD group were 19 (normal)/30 (abnormal), and 38.8%, respectively. The figures for the AD group were 10 (normal)/27 (abnormal), and 27.0%, respectively. The figures for the normal control group were 13 (normal)/16 (abnormal), and 44.8%, respectively. Their figures showed no statistically significant difference (Pearson χ2 = 2.409, *p* = 0.300) (Table 2). The results of the Titmus stereo fly test were not different between three groups.

**Table 2:** Associated results of the Titmus Stereo Fly Test, in idiopathic Parkinson disease patients, Alzheimer’s disease patients, and Controls.

Titmus fly test	IPD (n = 49)	AD(n = 37)	Controls(n = 29)	Pearson Chi-square value	*P* value
Normal (<60arcsec)	19 (39%)	10 (27%)	13 (45%)	2.409	*P* = 0.300
Abnormal (≥60arcsec)	30 (61%)	27 (73%)	16 (55%)		

IPD: Idiopathic Parkinson’s disease, AD: Alzheimer’s disease.

### Results from the stereopsis questionnaire

**Fig 2:**
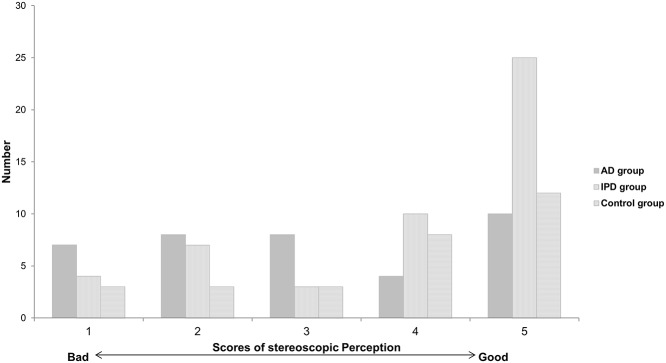
The number of patients, according to stereopsis questionnaire scores, in three groups. IPD: Idiopathic Parkinson’s disease, AD: Alzheimer’s disease.

**Fig 3:**
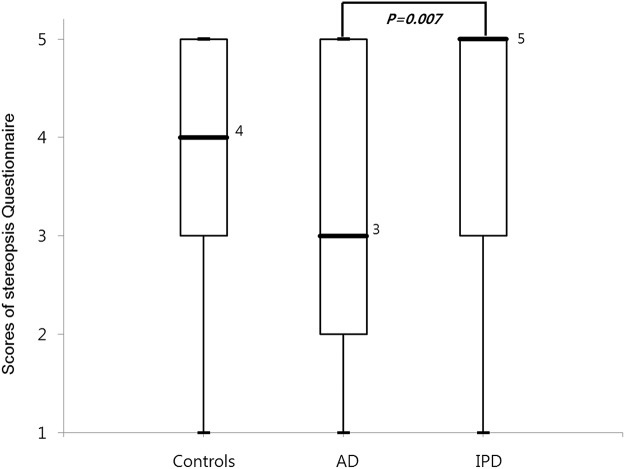
Scores of stereopsis questionnaire in three groups. Number: Median value. IPD: Idiopathic Parkinson disease, AD: Alzheimer’s disease.

The patients in each group were classified, and are presented, by their scores on the stereopsis questionnaire (Fig 2). Those who answered that they had no problem in their stereopsis accounted for the largest share in all three groups. For the other answers, the scores varied by each group. The median score of the stereopsis questionnaire were: 3 for the AD group, 5 for the IPD group, and 4 for the normal control group. The AD group showed statistically significant lower points, than other groups (*p* = 0.007) (Fig 3).

### Analysis on correlations and partial correlations between the scores on the stereopsis questionnaire, and other variables in each group

Correlations between the scores on the stereopsis questionnaire, and other variables (age, MoCA scores, Timus Stereo Fly Test) are shown in Table 3. No variables in the AD group had statistically significant correlation with the scores of the stereopsis questionnaire. However, the patients’ age and MoCA scores showed statistically significant relation in the IPD group. In the normal control group, only the Titmus Stereo Fly Test had a statistically significant relation, unlike other groups with degenerative disease. When the total three groups were combined, to increase the number of samples, all three variables showed statistically significant correlation. But the highest correlation coefficient was produced from the analysis on the MoCA scores. Analysis on the partial correlation coefficient was conducted, to identify the variable with the highest correlation with the scores of the stereopsis questionnaire, among age and MoCA scores that had been analyzed to have correlation with stereopsis in the combined group. When age was used as a control variable, the partial correlation coefficient between MoCA scores, and scores of the stereopsis questionnaire, showed statistically significant correlation (partial correlation coefficient: 0.319, *p* = 0.001). But when MoCA scores were used as a control variable, the correlation between age and stereopsis questionnaire scores was not statistically significant (partial correlation coefficient: 0.144, *p* = 0.127). Thus, MoCA scores were the most important variation to correlate with the stereopsis questionnaire.

**Table 3:** Correlations of stereopsis questionnaire scores, and Age, MoCA and the Titmus stereo Fly Test.

Correlation coefficient of stereopsis questionnaire scores	IPD(n = 49)	AD(n = 37)	Controls(n = 29)	Total(n = 115)
Age	-0.369**	0.155	-0.147	-0.242**
MoCA	0.308*	0.283	0.276	0.366**
Titmus Stereo Fly Test	-0.161	0.011	-0.625**	-0.209*

AD: Alzheimer’s disease, IPD: Idiopathic Parkinson’s disease.

Total: IPD+AD+Controls, Montreal cognitive assessment

* p<0.05

**p<0.01

## Discussion

Stereopsis or depth perception is the visual ability to perceive the world in three dimensions, and the distance of an object in the cerebrum, through information such as monocular cues, or binocular cues. Stereopsis created by binocular disparity, one of the binocular cues, is a complicated visual ability that exists only in humans, and in some higher animals. Stereopsis involves many factors, such as visual acuity, condition of the retina, optic nerve from the retina to the cerebrum, thalamus or basal ganglia, striate cortex in the occipital lobe, and visual association cortex and many other cortexes, etc. Since many nerve structures that are involved in stereopsis are tangled with each other, most studies on stereopsis have been confined to brain science. Most studies had been done on animals, instead of on humans.[34–38] Scientists have only recently begun studying diseases caused by impaired cranial nerves. However, since these studies were done only with tools that could measure binocular disparity, one of the binocular cues, it was difficult to identify the actual stereopsis of patients with neurodegenerative diseases. As mentioned in the introduction, 3D moving images shown through 3D television may be the closest visual stimulus to actual depth perception arising from monocular cues and binocular cues. Therefore, in this study, we have used a 3D TV to analyze how normal people, and patients with neurodegenerative diseases, perceive actual 3D moving images.

We have used the Titmus Stereo Fly Test, to identify differences among the IPD, AD groups and normal control group, in their stereopsis. In past studies, the IPD group showed different binocular disparity from that of the normal control group.[15] However, this did not happen in this study, because: those with normal binocular disparity (below 60 arcsec) accounted for 90% of the normal control group in the past study; but this time, only 44.8% of the participants in the group had normal binocular disparity. Studies that looked into binocular disparity in normal people have shown that the average binocular disparity of those in their 60s and 70s soared to 94 arcsec and 222 arcsec, making it difficult to find out the cutoff value for normal binocular disparity in elderly people.[39] Probably, the five-year age gap between the normal control group in the past study, and this study (average age in the past study and this study: 63 and 68, respectively) contributed to the little difference shown between the groups with degenerative diseases, and the control group, in this study. Although the age gap between the past and this study resulted in little differences among the three groups, using only binocular disparity, in measuring stereopsis in old-aged IPD and AD patients, can hardly be deemed appropriate.

Scores of stereopsis questionnaire from the AD group who were shown 3D images through 3D TV were significantly lower, than those of the IPD group, and the control group. In other words, the AD group was more decreased in their stereopsis, than the control or IPD group. It is well known that AD patients have decreased visuospatial function.[29] It also results in declined cognitive function, and limited ability to perceive monocular cues with the progress of the disease, which explains why they scored the lowest points, among the three groups. However, no correlation was found between the results from the cognitive function test, and the scores of the stereopsis questionnaire. It is very likely that the lack of correlation found between the MoCA scores and the scores of the stereopsis questionnaire stems from the fact that many parts of the cortical functions of the AD patients are decreased, which may affect their stereopsis.

Unlike the AD group, the IPD group scored statistically higher points in the stereopsis survey, than the AD patients, and showed little difference from the scores of the normal control group. The IPD group was in early- and mid-stage Parkinson disease. The degree of impairment in their cerebral cortex and cognitive functions were not serious enough to create difference from the normal control group, thereby showing no difference in their stereopsis. However, analysis on the correlation coefficient between their scores of stereopsis questionnaire and MoCA showed statistically significant correlation. In other words, IPD patients showed the most distinct correlation between the MoCA scores and questionnaire scores. First of all, lack of dopamine in their retina and other structures of the eye, weakened their visual function, resulting in declined stereopsis.[40] The dopaminergic pathway involves contrast sensitivity,[41] color vision,[42] motion perception,[43] etc. It controls retino-geniculate transmission, through D_1_ and D_2_ receptors that exist in the lateral geniculate body.[44] It also exists in the striate cortex, and visual association cortex.[45] Structures with dopaminergic pathway are essential, in perceiving stereoscopic vision. Therefore, lack of dopamine may have correlation with stereopsis. Second, the mesocortical dopaminergic pathway is involved in cognitive function, because it is involved in working memory and frontal executive function, producing a correlation between dopamine and cognitive function. These two reasons contribute to the positive correlation between the stereopsis scores and MoCA scores in IPD patients, as decreased dopamine weakens stereopsis and cognitive function in the patients.

What was interesting about the control group was that they showed strong negative correlation between their scores of stereopsis questionnaire, and Titmus Stereo Fly Test scores (correlation coefficient -0.625, p<0.01). This may mean that binocular disparity in normal people has much impact on their actual stereopsis, unlike IPD and AD patients, indicating that the Titmus Stereo Fly Test can be a meaningful tool, in measuring stereopsis in normal people. However, using only the Titmus Stereo Fly Test cannot be an appropriate tool to measure stereopsis in patients with neurodegerative diseases, as many of their cerebral structures that affect perception of contrast, relative size and height of the object, texture gradients, motion parallax, and accommodation, can easily be impaired.

When the number of samples was increased to 115 (AD, IPD and controls), the scores of the stereopsis questionnaire showed statistically significant correlation with age and MoCA scores. MoCA scores had higher correlation with scores of the stereopsis questionnaire, indicating that cognitive function has much impact on stereopsis. In order to find out which variable—age or MoCA scores—had stronger correlation, we have controlled for the effect of age and MoCA scores, to analyze the scores of the stereopsis questionnaire and correlation coefficient. This also showed that MoCA had higher correlation with the scores of the stereopsis questionnaire. When we analyzed the results with no regard to the characteristics of each disease, we could see that stereopsis of the actual images was affected more by cognitive function, than by age.

We have reviewed in this study that 3D images shown through 3D TV were more effective, than the several binocular disparity tests that had been used, in measuring stereopsis in elderly patients with neurodegenerative diseases. The results also showed that binocular disparity tests, such as the Titmus Stereo Fly Test, were effective in measuring stereopsis in the normal elderly, but had limits in analyzing actual stereopsis in patients with neurodegenerative diseases, such as IPD or AD. Thus, using 3D TV images that contain both binocular cues and monocular cues, and moving images seemed more appropriate in measuring stereopsis, in patients with neurodegenerative diseases.

In this study, it is weak point that AD patients were older than PD patients and Controls. Because onset age is different between AD with PD (AD occurs after 65 years, and PD occurs after 55 years), AD patients of mild to moderate stage are older than PD patients of mild to moderate stage. We analyzed the correlation and partial correlation between the scores of stereopsis questionnaire with age and MoCA score, found that the scores of stereopsis questionnaire were affected more by cognitive function, than by age.

This study could not find out which cognitive function affected the actual stereopsis the most, because only selective tests were conducted for the study, giving rise to the need for a follow-up study. Instead of the subjective questionnaires in this study, a more objectively scored program needs to be developed, to measure human stereopsis with 3D TV. The results from this study may be used in other studies, to prove actual differences in stereoscopic vision, in patients with degenerative diseases.

## Conclusion

This study showed that the results from binocular disparity tests used in past studies were different from the actual stereopsis in patients with neurodegenerative diseases, and that cognitive function was one of the factors that affected their stereopsis. We need to develop test tools, such as 3D TV, to measure stereopsis in patients more accurately, and to come up with appropriate treatments.
